# Does a specialized neurological ICU have better performance when compared with a general ICU?

**DOI:** 10.1186/cc14691

**Published:** 2015-09-28

**Authors:** Bruno F Mazza, Maria Fernanda Z Gatti, Rita de Cássia R de Macedo, Rosa GA Rocha, Samantha L Almeida

**Affiliations:** 1 Hospital Samaritano, Higienópolis, São Paulo, SP, Brazil

## Introduction

The costs of ICUs are high. American data show that they represent about 13 % of hospital costs, 4 % of the amount allocated to national health and 0.66 % of US GDP. Thus, it is necessary to use the best possible resource seeking the best for the institution and for patients. Studies show that a specialized unit in the treatment of neurological patients (ICU-N) has better results when compared with general ICUs (ICU-G).

## Objective

The objective of this study is to compare the results of patients with hospitalization for clinical neurological disorders in a neurological ICU with patients with the same diseases in general units.

## Methods

A retrospective analysis was performed from June to December 2014, using the database EPIMED^®^. All patients who were hospitalized with primary and secondary diagnoses of neurologic disorders were evaluated. SAPS severity score, length of stay and outcomes, and epidemiological profiles were analyzed. The results were descriptive and the percentage of cases, mean and standard deviation in the groups were analyzed.

## Results

Neurological disorders accounted for 17 % of admissions to the general units, against 94 % in the specialized unit. The mean age (ICU-N: 65 ± 15.16 vs. ICU-G: 63 ± 20.28) and sex (ICU-N: 45 % vs. ICU-G: 50 % male) were similar in both units. The most common diseases in the units were ischemic stroke, seizures, subarachnoid hemorrhage, intracerebral hemorrhage and transient ischemic attack. The average length of stay in the unit (7.29 ± 9.60 vs. 12.56 ± 21.22 days) and hospital (ICU-N: 14.39 ± 24.21 vs. ICU-G: 22.11 ± 43, 12 days) was lower in the ICU-N, and 78 % of patients had shorter hospital stays than 7 days, compared with 67 % in the ICU-G. The severity as measured by the SAPS 3 score was greater in ICU-G compared with the ICU-N (52.81 ± 16.05 vs. 49.53 ± 10.74) with a higher expected probability of death (26 68 % vs. 20.01 %). On the other hand, the ICU-N hospital mortality observed (6.52 % vs. 33.58 %) and thus the standardized mortality rate (0.33 vs. 1.36) was lower.

## Conclusion

The data relating to gender, age and diseases are similar in both units. The severity of disease is higher in the ICU-G. However, when analyzing the length of stay and mortality, the specialized unit has better performance, reducing the length of stay in the ICU and in the hospital and showing a significant difference in mortality.

